# Phenotypic flexibility of gape anatomy fine-tunes the aquatic prey-capture system of newts

**DOI:** 10.1038/srep29277

**Published:** 2016-07-07

**Authors:** Sam Van Wassenbergh, Egon Heiss

**Affiliations:** 1 Department of Biology, Universiteit Antwerpen, Universiteitsplein 1, B-2610 Antwerpen, Belgium; 2 Evolutionary Morphology of Vertebrates, Ghent University, K.L. Ledeganckstraat 35, B-9000 Gent, Belgium; 3 Departement d’Ecologie et de Gestion de la Biodiversité, Muséum National d’ Histoire Naturelle, 57 rue Cuvier, Case postale 55, 75231, Paris Cedex 5, France; 4 Institute of Systematic Zoology and Evolutionary Biology, Friedrich-Schiller-University Jena, Erbertstraße 1, 07743 Jena, Germany

## Abstract

A unique example of phenotypic flexibility of the oral apparatus is present in newts (Salamandridae) that seasonally change between an aquatic and a terrestrial habitat. Newts grow flaps of skin between their upper and lower jaws, the labial lobes, to partly close the corners of the mouth when they adopt an aquatic lifestyle during their breeding season. Using hydrodynamic simulations based on μCT-scans and cranial kinematics during prey-capture in the smooth newt (*Lissotriton vulgaris*), we showed that this phenotypic flexibility is an adaptive solution to improve aquatic feeding performance: both suction distance and suction force increase by approximately 15% due to the labial lobes. As the subsequent freeing of the corners of the mouth by resorption of the labial lobes is assumed beneficial for the terrestrial capture of prey by the tongue, this flexibility of the mouth fine-tunes the process of capturing prey throughout the seasonal switching between water and land.

Many animals benefit from reversible phenotypic plasticity in response to seasonal variation in the environment. Well-documented cases of such phenotypic flexibility (*sensu* Piersma & Drent^1^) are, for example, subcutaneous fat deposits anticipating to forthcoming high energy demands of migration in birds^2^,^3^,^4^ or bats^5^, hair growth to decrease thermal conductance of a mammal’s fur in winter^6^,^7^, and anatomical changes in specific brain regions controlling the production of a songbird’s singing behavior in the breeding season^8^,^9^. Phenotype flexibility is also documented in the morphology of the food-uptake system of animals in response to seasonal changes in diet: for example, gut length adapts to the digestive requirements of the available food in many vertebrates^10^,^11^,^12^, and relative growth of the jaws and shell of sea urchins changes during a year in response to changes in available food^13^. Yet, these reversible forms of phenotypic plasticity are much less common than non-reversible, developmental phenotypic plasticity^1^.

Probably one of the most drastic seasonal changes in life-style within vertebrates is found in newts, a subgroup within the Salamandridae. Adult newts become aquatic in spring when their breeding season starts, and leave the water near the end of the summer to live on land until next spring^14^. A substantial phenotypic flexibility related to these life stages allows us to distinguish a terrestrial and aquatic morphotype within each species: when newts become aquatic they develop tail fins, whereas the skin becomes more keratinised when they become terrestrial^15^,^16^. Also the morphology of the cranial system changes reversibly: flaps of skin grow from the upper jaw down to the lower jaw and partly closes the mouth corners like a curtain at the onset of the newt’s aquatic phase^15^ (Fig. 1). These labial fringes of skin sealing part of the left and right corners of the mouth are generally referred to as the labial lobes^17^.

In contrast to the terrestrial capture of prey by tongue prehension, newts generate suction to capture prey during their aquatic phase^18^,^19^. By restricting the gape to a narrower rostral opening, the labial lobes are hypothesised to increase the velocity of water flow just in front of the mouth when newts are suction feeding^20^ by limiting the amount of (less useful) flow curling into the mouth cavity from the lateral sides^21^. The fact that labial lobes occluding the lateral gape are never present in amphibians that do not use suction feeding^22^ suggests an important role of lateral gape occlusion for suction feeding. It was shown experimentally that a flatter shape of an opening surface (as with a circular mouth aperture) allows a faster outflow of water from a large container under steady flow conditions compared to wedged, open mouth-corner like outflow openings^23^. Computational model simulations under more realistic, unsteady flow conditions confirmed that the closing of the corners of the mouth helps to concentrate the flow at the centre of the mouth where suction feeders generally position their prey^24^. In many suction-feeding fishes, a temporal occlusion of the mouth corners is realised by the forward swinging of the left and right maxillary bones, a movement mechanically coupled with the separation of the upper and lower jaw^25^,^26^. Consequently, the generality of some type of occlusion of the lateral gape in suction feeders, together with the abovementioned hydrodynamic modelling studies, suggest an important improvement of suction-feeding performance by this morphological trait.

However, to which extent the incomplete occlusion of the mouth corners during the transition from the terrestrial to the aquatic morphotype in newts (Fig. 1) improves suction-feeding performance remains unknown. A recent study showed that a fully aquatic salamander (*Andrias davidianus*) is a remarkably fast and powerful suction feeder despite its almost fully open mouth corners^27^. In addition, flow visualisation experiments showed that immobilizing the maxilla of a fish (*Amia calva*) did not result in decreased flow velocities in front of the mouth, perhaps due to behavioural compensation of the fish that responded by opening the mouth wider and longer^28^. The goal of the present study is to unravel the functional implications of the phenotypic flexibility in the cranial system by quantifying the presumed hydrodynamic advantage of the labial lobes for suction feeding in the smooth newt (*Lissotriton vulgaris*).

## Results

The effect of the presence of labial lobes on the hydrodynamics of suction feeding in *Lissotriton vulgaris* was analysed by mathematical simulations of suction flows, using the numerical modelling technique of computational fluid dynamics (CFD). These simulations used realistic shapes of the external head and oropharyngeal cavity surfaces obtained from μCT scanning of *L. vulgaris* specimens in a resting position with a very small mouth opening to serve as the starting configuration of the model (Fig. 2). Programming of the movements and deformations of the head surfaces that expand the oropharyngeal cavity (and thereby generate suction) was based on kinematic data from high-speed videos of the newts during their aquatic capture of prey^19^ (Fig. 2a–c). Results from CFD are then compared between three models (Fig. 2e): one with the labial lobes (*model 1*), one without the labial lobes expanding at the same speed as model 1 (*model 2*), and one without the labial lobes expanding at an increases speed so that the peak power requirement (Fig. 2d) is equal to that of model 1 (*model 3*).

At the midsagittal plane, the presence of the labial lobes significantly increases the velocities of the flows of water directed to the back of the oropharyngeal cavity. At four points distributed along a central line on this medial plane (points p1 to p4; Fig. 3), the peak anterior-to-posterior flow velocity is consistently higher in model 1 (aquatic morph, blue curves in Fig. 3) compared to two models without the labial lobes (terrestrial morph; red and green curves in Fig. 3). Both the absolute and relative difference increases from points more in front of the mouth to the points more inside of the mouth (i.e., from p1 to p4). At 3 mm in front of the snout tip (p1 in Fig. 3), peak flow velocity of model 1 was 30% higher than in model 2, and 10% higher than in model 3. At 4.5 mm from the snout tip inside the mouth (p4 in Fig. 3), this difference increased to 75% with model 2, and 56% with model 3.

However, at the lateral side of the mouth, the two models of the terrestrial morph with the fully open lateral gape still generated anterior-to-posterior flow for the point located just anterior of the mouth corners of these two models (p5 in Fig. 3), but no longer when the labial lobes are added. Also further posterior on the lateral side, at the level of the eye, considerably stronger suction flows occur in the models without the labial lobes (p6 in Fig. 3). At this point p6 (Fig. 3), which is located at the lateral extension of the mouth corners of models 2 and 3, the flow is directed medio-anteriorly. The large difference between the models in the flow patterns at the lateral sides of the head is also clearly illustrated in Fig. 4a–c (see also Supplementary Movie 1).

Iso-velocity surfaces show that suction flows reach further in front of the mouth in the model with the labial lobes (Fig. 4d,e). Anterior-to-posterior flow of 2.5 mm s^−1^ is found at 5.6 mm in front of the snout tip in model 1, which is 15.5% further than model 2 and 9.4% further than model 3. A similar relative difference is found for the 10 mm s^−1^ iso-velocity surface that reached 17.7% and 9.0% further in model 1 compared to, respectively, model 2 and 3. A broader spreading of the generated flow field at the lateral side of the head for the models without the labial lobes (models 2 and 3; red and green curves) is also evident from the iso-velocity lines (Fig. 4e).

Two more direct measures of suction performance were also compared between the models. Firstly, the distance from which a small, neutrally buoyant prey particle (assumed to follow the water flow) can be sucked to reach a given location inside the mouth at the level of the eye by the end of the simulation was calculated. The model with the labial lobes outperformed the other two models by moving the prey over a 15.2% longer distance (Fig. 5a). Secondly, the force exerted on a fixed prey was evaluated by adding a sphere of 0.5 mm diameter just in front of the mouth in all models and numerically calculating the total hydrodynamic force (pressure and shear force) on the mesh of the sphere at each time step of these new simulations. Also here the model with the labial lobes (model 1) showed the best performance with a peak force of 14.6 μN, which is 46.8% higher than model 2 and 16.8% higher than model 3 (Fig. 5b).

## Discussion

**Figure 1:**
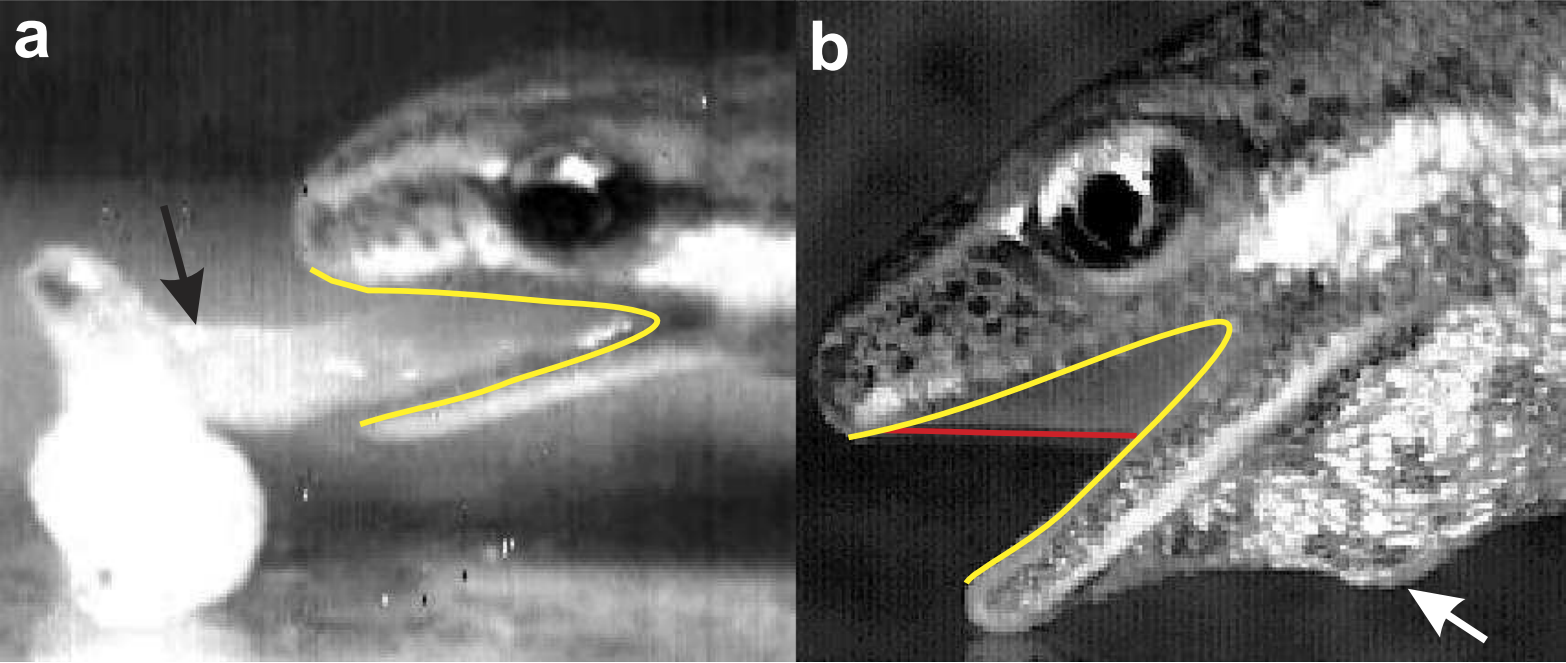
Feeding modes and anatomical flexibility of the gape in *Lissotriton vulgaris*. Protrusion and prey-adhesion by the tongue (black arrow) is used during the terrestrial phase when the lateral side of the gape (yellow outline) is open (**a**). During the newt’s aquatic phase (**b**), suction produced mainly by depression of the hyoid (white arrow) is used to draw prey into the oropharyngeal cavity. Labial lobes (anterior outline in red) close the mouth corners during this aquatic phase.

The well-documented metamorphosis of larval salamanders accompanying their transition to a terrestrial lifestyle is manifested in nearly all of their organ systems^29^, including the cranial components of the feeding system^30^. After metamorphosis, in many cases the feeding system drastically changes from a design to capture prey underwater by generating suction, to a system relying on tongue projection from the mouth. In the tiger salamander (*Ambystoma tigrinum*), for example, not only the tongue and its supporting musculoskeletal system develops during metamorphosis, but also the well-developed labial lobes on the upper and lower jaws reduce to form a more open, notched lateral gape with a posteriorly shifted point of articulation^30^. Since the labial lobes are not only reduced during the aquatic-to-terrestrial transition during metamorphosis but are also lost and regained yearly on the upper jaw in newts that seasonally transition between water and land^15^ (Fig. 1), it is important to understand the functional consequences of this feature.

Our analysis showed that the phenotypic flexibility of the re-growth of the labial lobes significantly increases the suction-feeding performance of the smooth newt (*L. vulgaris*). Peak flow velocities at the centerline of the oropharyngeal cavity increased between 10% and 50% depending on the location (Fig. 3), enabling prey to be sucked from 15% farther (Fig. 5a). As these newts mainly feed on benthic insects and oligochaetes that often adhere to the substrate, also the capacity to dislodge such fixed prey by suction improves due to a 17% increase in peak suction force (Fig. 5b). These results confirm the hypothesis that lateral gape occlusions are beneficial for suction feeding^21^,^22^,^23^,^24^,^31^. However, previous reports stating that the labial lobes are critical for effective suction feeding^17^ are probably exaggerated given the less than 20% difference in suction performance between models with and without the labial lobes. Nevertheless, our results strongly suggest that the seasonal growth and resorption of the labial lobes is an adaptation to seasonally changing prey capture conditions.

A logical question is then what the disadvantages are for retaining the labial lobes during terrestrial life. It is assumed that the loss of the labial lobes allow the gape to be opened wide enough for the unobstructed tongue projection and subsequent capture and manipulation of large active terrestrial prey with the jaws^30^. In addition to this, we hypothesise that a narrowing of the mouth aperture is less problematic during aquatic feeding because suction flows tend to align prey parallel with the streamlines that predominantly enter straight into the mouth^32^. As an automatic (flow-driven) reorientation of the prey will not take place when, for example, a terrestrial worm adhered to the tongue is pulled back into the mouth, the worm may be lost during contact with the anterior edges of the labial lobes.

Potential interference with the maximal size of prey to fit into the mouth may also explain why the labial lobes do not grow even further towards the tip of the jaws in *L. vulgaris* (Fig. 1). Anterior of the eyes, the edges of the jaws start curving medially. Therefore, additional growth of the labial lobes from this point forward would result in a reduced width of the mouth opening. Such a trade-off between lengthening of the oropharyngeal cavity by growth of the labial lobes (improving suction performance) and mouth width (limiting maximal prey size) is not present closer to the jaw joint where curving of the jaws towards the medial plane is negligible. As diet choice experiments indicated that *L. vulgaris* has a selective preference for large-sized prey^33^, gape size may limit foraging efficiency in this species.

**Figure 2:**
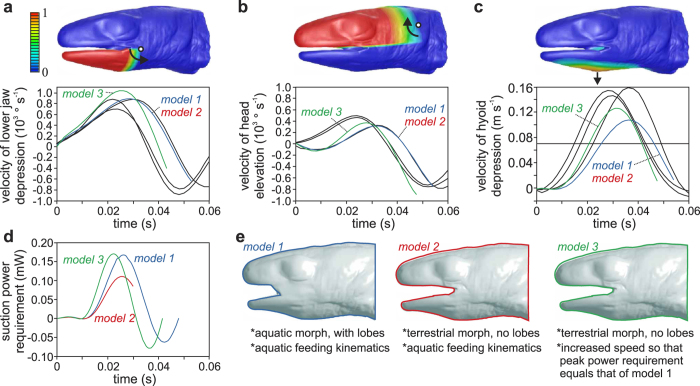
Kinematic input and power requirement of the three models. The velocity of three movements are used as input in the models: lower jaw depression (**a**), head elevation (**b**), and hyoid depression (**c**). *In-vivo* kinematics of three aquatic feeding acts are shown (black curves) alongside the kinematic input of the models (coloured curves). Each kinematic input variable is applied only to the relevant zones of the mesh, as shown in the coloured images at the top: a factor of 1 of the mapped colour scale (red) means that the equation for this kinematic variable is followed for 100%, a factor of 0 (blue) means that the mesh remains unaltered. Power requirements of the three models are shown in (**d**). The properties of models 1, 2, and 3 with their respective colour coding used in this article are shown in (**e**).

Our hydrodynamic simulations indicate a drawback to the closing of the lateral gape: the duration of the expansion of the head will inevitably increase if we assume no change in the power input from the feeding muscles. This result was evident from our power-normalisation procedure that predicted that the labial lobes will cause the mouth and hyoid expansion to last 15% longer due to power demands (Fig. 2d). Despite that stronger flows will be created, this inevitably means a longer gape cycle, and therefore more time for agile prey to start an attempt to escape. As the labial lobes elongate the buccal cavity, this effect will probably be prominent in animals with very long mouth cavities. Extremely long mouth cavities are found in syngnathid fishes (e.g. pipefishes, seahorses). Notably, syngnathids have evolved a mechanism based on elastic recoil of tendons to amplify the power for suction feeding, which ensures a sufficiently fast buccal expansion^34^. The temporarily closing of the mouth corners by the forward swinging of the maxillary bones, as observed in many ray-finned fishes, may be optimal to realize a fast gape cycle combined with far-reaching suction flows. However, how the time-varying shape of the mouth interacts with the dynamics of oropharyngeal expansion is not well understood^24^,^35^.

In conclusion, our study showed that the phenotypic flexibility in the growth/resorption of the labial lobes is an adaptive response to improve prey-capture performance. Even after accounting for the possibility that a faster oropharyngeal expansion can be performed with open mouth corners, suction-feeding performance increases notably due to the labial lobes. We argued that suction-flow mediated centering and straightening of the prey during aquatic feeding limits the spatial constraints of the reshaping of the mouth (i.e. adding of the labial lobes) on feeding on larger prey. As these spatial constrains are probably more prominent during the tongue-based, terrestrial capture of prey^30^, this may explain the loss of the labial lobes during the terrestrial phase.

## Methods

### CFD modelling strategy

Special attention was paid to include a normalisation of the power that is required to cause the movement of the different CFD models against the expansion-resisting hydrodynamic forces that are exerted at their surfaces. It is generally accepted that the speed of expansion of the oropharyngeal cavity of a suction feeder is determined by the amount of power available from the muscles of the suction-feeding apparatus^36^. However, the power requirement of an oropharyngeal expansion depends on the morphology of the oropharyngeal cavity^37^,^38^. Since removing of the labial lobes can be regarded as a shortening of the actual cavity of the oropharynx, and shorter cavities can perform faster radial expansion for a given power input^38^, newts with an open lateral gape but with the same musculature should probably be able to expand their head faster than ones with the lateral side of the gape occluded by the labial lobes. As *in-vitro* experiments have shown that the fast-fibred cranial and post-cranial muscles of suction feeders can generate their maximal power at a relatively broad range of muscle shortening speeds (e.g., >90 of maximal power of catfish hypaxials attached to the pectoral girdle for cyclical shortening between 15 and 25 Hz^39^), we assume that the power available from the muscles will be the same for changes in expansion speed of less than 25%. Thus, to account for the effect of the presence of the labial lobes on head expansion speed, the kinematics of the model without the lobes (*model 2*) was adjusted in a new simulation (*model 3*) that requires the same power as the model of the aquatic morph that does have the labial lobes (*model 1*).

### Model geometry and mesh

In preparation for μCT scanning, a male newt of the terrestrial morphotype was fixed in 4% formaldehyde. The animal used for the present study was collected between April-June 2011 in Lower Austria, Austria with collection permission RU5-BE-18/022-2011 granted by the local government of Lower Austria. All methods were approved by the Ethical Commission for Animal Experiments of the University of Antwerp (code: 2010-36). All procedures were conducted in accordance with their guidelines. After dehydration in a graded series of ethanol, the specimen was contrasted in a solution of 1% elemental iodine in absolute ethanol for two weeks to increase X-ray absorption by the soft tissues^40^. Next, the sample was rinsed in absolute ethanol for several hours and mounted in Falcon tubes again in absolute ethanol. A scan of the whole head was acquired using a SkyScan 1174 (Bruker, Belgium) micro CT scanner with a source voltage of 50 kV and a isovolumetric voxel resolution of 7.39 μm. To reconstruct the outer and inner surfaces of the head, the CT image stacks were processed with the 3D software package Amira 4 (FEI Visualization Sciences Group, Merignac Cedex, France). The output surfaces from Amira were first converted into a single, watertight surface using Geomagic Qualify 10 software (Geomagic Inc., Morrisville, North Carolina). Mimicking the *in-vivo* shape and position of the labial lobes during feeding (Fig. 1), surface connections were added in Geomagic between the upper and lower jaws to represent the labial lobes (Fig. 2e). Both the original and labial-lobed model surfaces were transformed into NURBS surface patches using VRMesh Studio 5.0 (VirtualGrid, Seattle, WA).

Next, the NURBS model of the salamander head was centered in a spherical boundary of the flow domain with a radius of 50 mm (ANSYS DesignModeler 14.5.7; ANSYS Inc., Canonsburg, Pennsylvania), and the space external of the head surfaces was meshed with approximately 6 million tetrahedral cells (size of about 50 μm at the head surfaces and 5 mm at the outer domain boundary and a growth rate of 1.1 between the two) in ANSYS Meshing 14.5.7. Computations of skewness (mean 0.22, maximum 0.85) and orthogonal quality (mean 0.86, minimum 0.17) indicated an acceptable quality of the tetrahedrons in the mesh. We monitored the dorsal force on the internal surface of the lower jaw at the simulation time of 25 ms during a series of mesh-convergence tests. Using a relatively coarse mesh (0.8 million tetrahedrons) it was first shown that changing the size of the spherical outer boundary did not influence the result (>0.3% change between radii of 30 and 100 mm). A range of meshes with 0.5, 0.8, 2.1 and 6.4 million tetrahedrons showed only −0.33% change during the final step of refinement. Therefore, we considered the 6 million tetrahedrons mesh to be sufficiently accurate.

### Solver settings and kinematic input

The spherical outer boundary was set as a pressure outlet, and the no-slip condition was enforced at the solid boundaries of the salamander head. The model was solved for unsteady, laminar flow in ANSYS Fluent 14.5.7 with a time step of 0.5 ms. Using a larger time step of 1 ms did not substantially influence the solution (e.g; only 1.1% difference in the monitored force), indicating that 0.5 ms was sufficiently small. Forty iterations per time step were sufficient to obtain iterative convergence. Since transition to turbulent flow is very unlikely to occur because of the short duration and high accelerations of the water during suction feeding^41^, a laminar flow model was chosen. The pressure-based solver (chosen to obtain fast-converging solutions) was used. The first-order implicit unsteady formulation option was used in the simulation because moving mesh simulations (see further) currently only work with first-order time advancement. The standard pressure discretisation scheme was used for the pressure calculation and a second-order upwind scheme was used for momentum equations. The pressure-velocity coupling was solved using the robust, default SIMPLE scheme. The latter is a discretisation method that uses a relationship between velocity and pressure corrections to enforce mass conservation and to obtain the pressure field. The physical properties assigned to the main fluid zone were that of normal water at 20 °C (density 998.2 kg m^−3^, viscosity 1.003 mPa s).

To match the prey-capture kinematics of *L. vulgaris* as close as possible, the motion and deformation of the mesh was prescribed by a user-defined function (DEFINE_GRID_MOTION; see the ANSYS Fluent UDF Manual version 14.5 published in 2012). To do so, representative profiles of three kinematical variables quantified in a previous study^19^ were accurately fitted with sixth-order polynomial functions: angular velocity of the lower jaw (Fig. 2a), angular velocity of the head (Fig. 2b), and linear velocity of hyoid depression (Fig. 2c). Since the hyoid depression as calculated from anatomical landmark displacements (i.e. eye to hyoid tip distance) in the high-speed videos also includes a small amount of posterior displacement while the mesh-motion function simplified this into a purely vertical motion, the fitted profile for hyoid-depression velocity was reduced by 30% (Fig. 2c). The three polynomial equations were applied to specific nodes of the mesh by using a factor between 0 (following the motion equation for 0%) to 1 (following the motion equation for 100%) that is stored in the user-defined node memory of ANSYS Fluent (Fig. 2a–c). The gradual decrease of these factors at the borders of the targeted region prevents mesh intersections from forming. The software was set to move all internal mesh nodes (spring-based smoothing algorithm; spring constant factor = 1; Laplace node relaxation = 1) and re-mesh (make new mesh for triangles smaller than 1 × 10^−5 ^m and larger than 5 × 10^−3 ^m) after every time step in response to the motion of the boundaries prescribed in the three polynomial equations included in the mesh-motion user-defined function.

To normalise the power input, the total power requirement of the model to overcome hydrodynamic resistance by the two morphs under the input of aquatic feeding kinematics (Fig. 2a–c) was first calculated. The instantaneous power requirement was determined by taking the scalar product of the force (opposing the pressure and shear force by the water) and velocity vector of each triangle, then summing these values for the entire head surface of the newt. The resulting peak power requirement for model 1 (aquatic morph) was 0.17 mW, and 0.11 mW for model 2 (terrestrial morph). By adjusting the input velocity to peak 17% higher, and the peak acceleration to become 35% higher in a 15% shorter simulation with equal expansion amplitude, model 3 was created as a power-normalised simulation for the terrestrial morphotype feeding underwater. A graphical representation of the three models is given in Fig. 2e. The variation in expansion time between the models implies that we assume that the neural control system of newts is capable of adjusting the activation timings of the muscles of the feeding system to a small degree (15% variation in activation duration with equal relative timing between lower jaw and hyoid depression).

**Figure 3:**
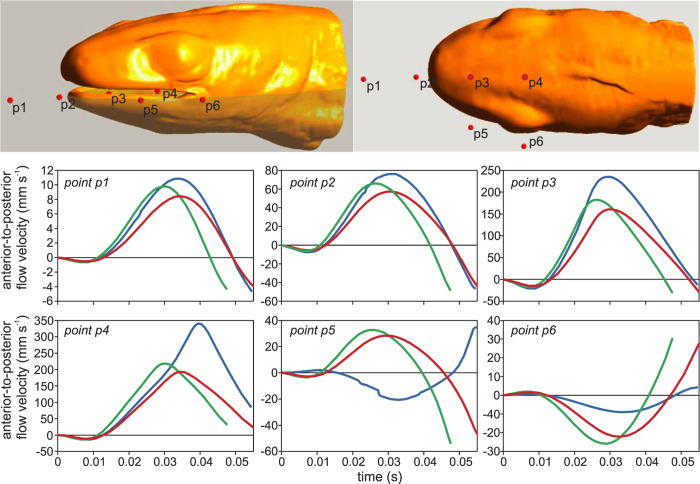
Suction-flow velocities along the anterior-to-posterior axis at six fixed locations. The six locations, points 1 to 6, are shown in the top images. The three colours represent the models (blue = model 1, green = model 2, red = model 3; Fig. 2e).

**Figure 4:**
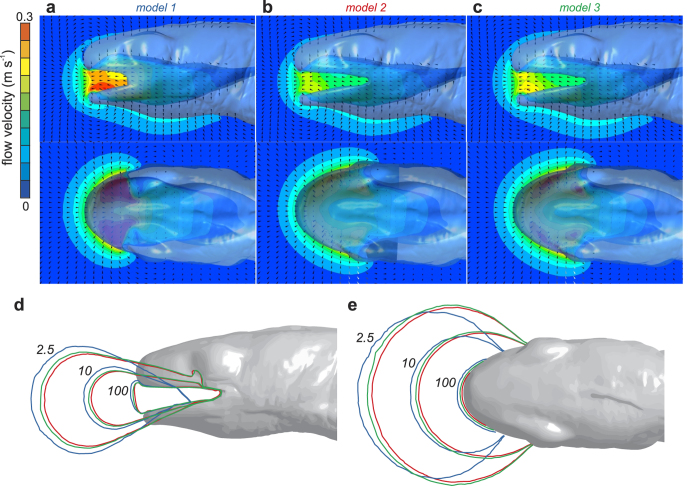
Comparison of suction-flow velocities. Three-dimensional flow velocities (colour scale on the left) and directions (small black arrowheads) are given for the midsagittal and midfrontal section planes of model 1 (**a**), model 2 (**b**), and model 3 (**c**) (Fig. 2e). Iso-contours of anterior-to-posterior flow velocity (2.5, 10, and 100 mm s^−1^) for the three models (see top or Fig. 2e for colour codes) are shown in (**d**,**e**). The displayed images are at an instant of about 3 ms before maximal flow velocity at the front of the mouth, and represents equal states of expansion for models 1 and 2 (simulation time = 27 ms) *versus* model 3 (simulation time = 23 ms).

**Figure 5:**
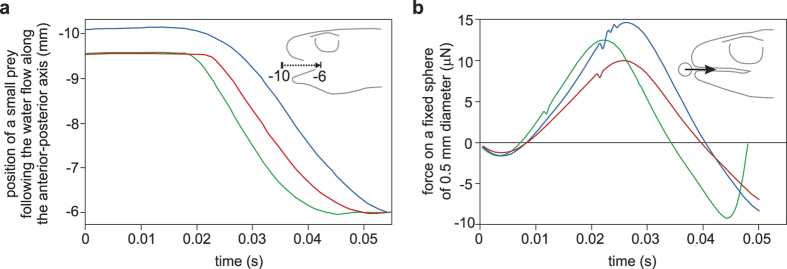
Comparison of prey transport and force-generating performance. In (**a**) the theoretical paths travelled by a small, neutrally buoyant prey following the water motion along the anterior-to-posterior axis to end up inside the mouth at the level of the anterior orbits at the end of the simulation. In (**b**) the total force (pressure force and shear force added) exerted on a stationary sphere with a diameter of 0.5 mm located just in front of the mouth. The three colours represent the models (blue = model 1, red = model 2, green = model 3; Fig. 2e).

A comparison of the flow velocity calculated by CFD at the most posterior point in between the jaws that is still visible from a lateral view (p3 on Fig. 3) with peak velocity of prey (i.e. relatively large maggots) measured on high-speed videos^19^ (*N* = 11) was used to determine whether the model output is realistic. The mean (±s.d.) peak velocities of the prey was 0.182 ± 0.036 m s^−1^. CFD predicted an instantaneous peak velocity of 0.235 m s^−1^, which is within the 95% prediction range interval of the *in-vivo* measurements. This shows that the calculated flow velocity magnitudes are realistic. Yet, at the final instants of the simulation (during mouth closure), some water starts to flow anteriorly out of the mouth. These final instants may not have been accurately modelled, as some passive expansion further down the pharynx is probably taking place. However, this will hardly influence the presented performance comparisons, as the simulated prey paths are virtually unaffected by this local anterior flow, and the instant of peak force on attached prey is much earlier.

## Additional Information

**How to cite this article**: Van Wassenbergh, S. and Heiss, E. Phenotypic flexibility of gape anatomy fine-tunes the aquatic prey-capture system of newts. *Sci. Rep.* **6**, 29277; doi: 10.1038/srep29277 (2016).

## Supplementary Material

Supplementary Movie Legend

Supplementary Movie 1

## References

[b1] Piersma T. & Drent J. Phenotypic flexibility and the evolution of organismal design. TREE 18, 228–233 (2003).

[b2] Bairlein F. Body weights and fat deposition of Palaearctic passerine migrants in the central Sahara. Oecologia 66, 141–146 (1985). 10.1007/BF00378566 28310826

[b3] Guglielmo C. G. & Williams T. D. Phenotypic flexibility of body composition in relation to migratory state, age and sex in the western sand-piper. Physiol. Biochem. Zool. 76, 84–98 (2003). 12695989 10.1086/367942

[b4] Wu M., Xiao Y., Yang F., Zhou L., Zheng W. & Liu J. Seasonal variation in body mass and energy budget in Chinese bulbuls (*Pycnonotus sinensis*). Avian Research 5, 4 (2014). 10.11813/j.issn.0254-5853.2014.1.033 PMC5042950 24470452

[b5] McGuire L. P., Fenton M. B. & Guglielmo C. G. Phenotypic flexibility in migrating bats: seasonal variation in body composition, organ sizes and fatty acid profiles. J. Exp. Biol. 216, 800–808 (2013). 23408801 10.1242/jeb.072868

[b6] Harris G. D., Huppi H. D. & Gessaman J. A. The thermal conductance of winter and summer pelage of *Lepus californicus*. J. Therm. Biol. 10, 79–81 (1985).

[b7] Boyles J. G. & Bakken G. S. Seasonal changes and wind dependence of thermal conductance in dorsal fur from two small mammal species (*Peromyscus leucopus* and *Microtus pennsylvanicus*). J. Therm. Biol. 32, 383–387 (2007).

[b8] Nottebohm F. A brain for all seasons: cyclical anatomical changes in song control nuclei of the canary brain. Science 214, 1368–1370 (1981). 7313697 10.1126/science.7313697

[b9] Soma K. K., Tramontin A. D., Featherstone J. & Brenowitz E. A. Estrogen contributes to seasonal plasticity of the adult avian song control system. J. Neurobiol. 58, 413–422 (2004). 14750153 10.1002/neu.10288

[b10] Starck J. M. & Rahmaan G. H. A. Phenotypic flexibility of structure and function of the digestive system of Japanese quail. J. Exp. Biol. 206, 1887–1897 (2003). 12728010 10.1242/jeb.00372

[b11] Ke Z., Xie P. & Longgen G. Phenotypic plasticity in gut length in the planktivorous filter-feeding silver carp (*Hypophthalmichthys molitrix*). The Scientific World Journal 8, 169–175 (2008). 18301818 10.1100/tsw.2008.37 PMC5848700

[b12] Karasov W. H., Martínez del Rio C. & Caviedes-Vidal E. Ecological physiology of diet and digestive systems. Annu. Rev. Physiol. 73, 69–93 (2011). 21314432 10.1146/annurev-physiol-012110-142152

[b13] Ebert T. A., Hernández J. C. & Clemente S. Annual reversible plasticity of feeding structures: cyclical changes of jaw allometry in a sea urchin. Proc. R. Soc. B. 281, 20132284 (2014). 10.1098/rspb.2013.2284 PMC3924062 24500161

[b14] Nöllert A. & Nöllert C. Die Amphibien Europas. (Franckh-Kosmos Verlag, 1992).

[b15] Matthes E. Bau und Funktion der Lippensäume wasserlebender Urodelen. Z. f. Morphol. u. Ökol. d. Tiere 28, 155–169 (1934).

[b16] Warburg M. R. & Rosenberg M. Ultrastructure of ventral epidermis in the terrestrial and aquatic phases of the newt *Triturus vittatus* (Jenyns). Ann. Anat. 179, 341–347 (1997). 9272218 10.1016/S0940-9602(97)80073-8

[b17] Deban S. & Wake D. Aquatic feeding in salamanders. In Feeding: form, function and evolution in tetrapod vertebrates (ed. Schwenk K. ) 65–94 (Academic Press, 2000).

[b18] Heiss E., Aerts P. & Van Wassenbergh S. Masters of change: seasonal plasticity in the prey-capture behavior of the Alpine newt *Ichthyosaura alpestris* (Salamandridae). J. Exp. Biol. 216, 4426–4434 (2013). 24259258 10.1242/jeb.091991

[b19] Heiss E., Aerts P. & Van Wassenbergh S. Flexibility is everything: prey capture throughout the seasonal habitat switches in the smooth newt *Lissotriton vulgaris*. Org. Divers. Evol. 15, 127–142 (2015). 26097413 10.1007/s13127-014-0187-1 PMC4470538

[b20] Deban S. M. Constraint and convergence in the evolution of salamander feeding. In Vertebrate Biomechanics and Evolution (eds Gasc J.-P., Casinos, & Bels V. ) 163–180 (BIOS Scientific Publishers, 2003).

[b21] Muller M. & Osse J. W. M. Hydrodynamics of suction feeding in fish. Trans. Zool. Soc. Lond. 37, 51–135 (1984).

[b22] O’Reilly J. C., Deban S. M. & Nishikawa K. C. Derived life history characteristics constrain the evolution of aquatic feeding behavior in adult amphibians. In Topics in Functional and Ecological Vertebrate Morphology (Eds. Aerts P. & D’Août K. ) 153–190 (Shaker Publishing, 2002).

[b23] Lauder G. V. Feeding mechanics in primitive teleosts and in the halecomorph fish *Amia calva*. J. Zool. 187, 543–578 (1979).

[b24] Skorczewski T., Cheer A. & Wainwright P. C. The benefits from flat circular mouths on suction feeding performance. J. R. Soc. Interface 9, 1767–1773 (2012). 22319101 10.1098/rsif.2011.0904 PMC3385762

[b25] van Dobben W. H. Über den Kiefermechanismus der Knochenfische. Arch. Néerl. Zool. 2, 1–71 (1935).

[b26] Gosline W. A. Jaw structures and movements in higher Teleostean fishes. Jpn. J. Ichthyol. 34, 21–32 (1987).

[b27] Heiss E., Natchev N., Gumpenberger M., Weissenbacher A. & Van Wassenbergh S. Biomechanics and hydrodynamics of prey capture in the Chinese giant salamander reveal a high-performance jaw-powered suction mechanism. J. R. Soc. Interface 10, 20121028 (2013). 23466557 10.1098/rsif.2012.1028 PMC3627076

[b28] Sanford C. P. J., Day S. & Konow N. The role of mouth shape on the hydrodynamics of suction feeding in fishes. Integr. Comp. Biol. 49, E149 (2009).

[b29] Latimer H. B. & Roofe P. G. Weights and linear measurements of the body and organs of the tiger salamander, before and after metamorphosis, compared with the adult. Anat. Rec. 148, 139–147 (1964). 14123498 10.1002/ar.1091480204

[b30] Reilly S. M. & Lauder G. V. Metamorphosis of cranial design in tiger salamanders (*Ambystoma tigrinum*): a morphometric analysis of ontogenetic change. J. Morphol. 204, 121–137 (1990). 10.1002/jmor.1052040202 29865725

[b31] Miller B. T. & Larsen J. H. Feeding performance in aquatic postmetamorphic newts (Urodela, Salamandridae)–are bidirectional flow systems necessarily inefficient? Can. J. Zool. 67, 2414–2421 (1989).

[b32] Lauder G. V. Jr. Intraspecific functional repertoires in the feeding mechanism of the characoid fishes *Lebiasina*, *Hoplias* and *Chalceus*. Copeia 1981, 154–168 (1981).

[b33] Ranta E. & Nuutinen V. Foraging by the smooth newt (*Triturus vulgaris*) on zooplankton: functional responses and diet choice. J. Anim. Ecol. 54, 275–293 (1985).

[b34] Van Wassenbergh S., Strother J. A., Flammang B. E., Ferry-Graham L. A. & Aerts P. Extremely fast prey capture in pipefish is powered by elastic recoil. J. R. Soc. Interface 5, 285–296 (2008). 17626004 10.1098/rsif.2007.1124 PMC2607401

[b35] Day S. W., Higham T. E., Holzman R. & Van Wassenbergh S. Morphology, kinematics, and dynamics: the mechanics of suction feeding in fishes. Integr. Comp. Biol. 55, 21–35 (2015). 25980568 10.1093/icb/icv032

[b36] Carroll A. M. & Wainwright P. C. Muscle function and power output during suction feeding in largemouth bass. Micropterus salmoides. Comp. Biochem. Physiol. A 143, 389–399 (2006). 10.1016/j.cbpa.2005.12.022 16458031

[b37] Van Wassenbergh S., Aerts P. & Herrel A. Hydrodynamic modeling of aquatic suction performance and intra-oral pressures: limitations for comparative studies. J. R. Soc. Interface 3, 507–514 (2006). 16849247 10.1098/rsif.2005.0110 PMC1664642

[b38] Roos G., Van Wassenbergh S., Aerts P., Herrel A. & Adriaens D. Effects of snout dimensions on the hydrodynamics of suction feeding in juvenile and adult seahorses. J. Theor. Biol. 269, 307–317 (2011). 21029742 10.1016/j.jtbi.2010.10.023

[b39] Van Wassenbergh S., Herrel A., James R. S. & Aerts P. Scaling of contractile properties of catfish feeding muscles. J. Exp. Biol. 210, 1183–1193 (2007). 17371917 10.1242/jeb.000109

[b40] Metscher B. D. MicroCT for comparative morphology: simple staining methods allow high-contrast 3D imaging of diverse non-mineralized animal tissues. BMC Physiol. 9, 11 (2009). 19545439 10.1186/1472-6793-9-11 PMC2717911

[b41] Van Wassenbergh S. & Aerts P. Aquatic suction feeding dynamics: insights from computational modelling. J. R. Soc. Interface. 6, 149–158 (2009). 18782720 10.1098/rsif.2008.0311 PMC2575385

