# Supplementary material for: Phenotypic flexibility of gape anatomy fine-tunes the aquatic prey-capture system of newts
Source: Sci Rep. 2016 Jul 7;6:29277. doi: 10.1038/srep29277 (PMC4935879; doi:10.1038/srep29277)
Supplement: Supplementary Movie Legend [file srep29277-s1.doc]

Title: Phenotypic flexibility of gape anatomy fine-tunes the aquatic prey-capture system of newts

Authors: Sam Van Wassenbergh, Egon Heiss

**Supplementary Materials:** Supplementary Movie 1 (.mov 3.2 MB): Animation comparing the patterns of the flow generated by the three models.
